# Don't be scared: insert a mesh!

**DOI:** 10.4314/pamj.v10i0.72228

**Published:** 2011-10-10

**Authors:** Alain Chichom Mefire, Marc Leroy Guifo

**Affiliations:** 1 Faculty of Health Sciences, University of Buea and Regional Hospital Limbe, Limbe, Cameroon; 2 Faculty of Medicine and Biomedical Sciences, University of Yaoundé 1, Yaoundé, Cameroon

**Keywords:** Mesh, hernia, abdomen, surgery, Cameroon

## Abstract

Mesh repair is now the gold standard technique of repair on incisional hernias. Infection of the mesh is a challenging complication of this type of repair. The risk of mesh infection has been shown to be greater in case of complicated hernia. We present the case of a 64 years old female who presented with an incarcerated incisional hernia with bowel infarction. Treated with a non absorbable mesh repair, she developed mesh infection. The infection was successively treated with simple drainage. This case and review of relevant literature seem to be an indication that mesh repair could still be considered in cases of complicated hernia. Simple drainage usually helps manage the cases of mesh infection.

## Introduction

Synthetic mesh has revolutionized abdominal wall hernia repair. Mesh hernioplasty is now the standard technique of repair all large abdominal wall hernias, including incisional hernias. Infection of the mesh is one of the most challenging complications of this technique. It usually requires additional surgical procedures for debridement and sometimes the excision of the mesh. This complication is even more challenging in low income environment. Data on the outcome of mesh repair of incarcerated ventral hernia are scarce. We present the case of salvage of a mesh placed on an incarcerated giant incisional hernia.

## Case presentation

A 64 years old housewife is rushed in the emergency department for an acute diffuse abdominal pain of 12 hours duration, associated to vomiting and a localized abdominal distension. She had been diagnosed of a post-hysterectomy infra-umbilical incisional hernia two years earlier and was then proposed a suture repair, but could not have it done because of financial constrain.

**Figure 1:**
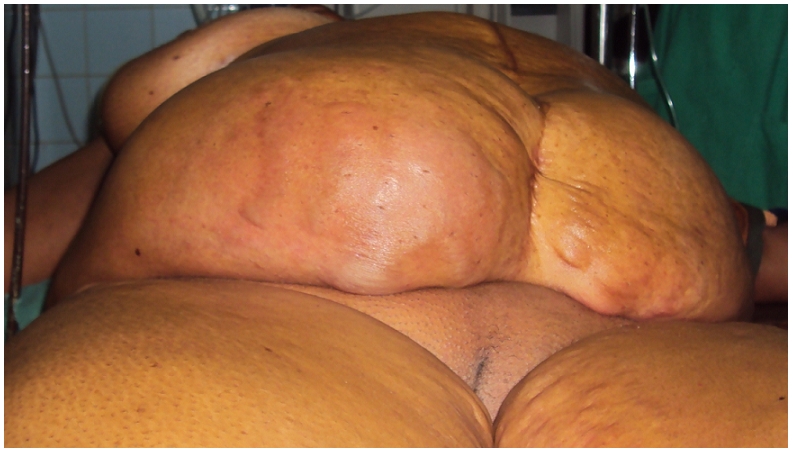
A pre-operative view of the abdomen of our patient showing the massive abdominal distension localized to the right side

On physical exam, the general condition is altered with a blood pressure of 90/50 millimetres of mercury and signs of moderate dehydration. There is an infra-umbilical midline scar on the abdomen. There is diffuse abdominal distension and a huge painful non reducible mass of the right side of the abdomen (Figure 1), exquisitely tender on palpation. They are signs of peritoneal irritation. Tympanic note is resonant and auscultation is silent. Pelvic exam is normal. A plain abdominal X-ray shows diffuse air-fluid levels. A full blood count displays a leucocytosis (14600/mm3) with shifting of the formula to the right (86% of neutrophils); the haemoglobin level is normal.

**Figure 2:**
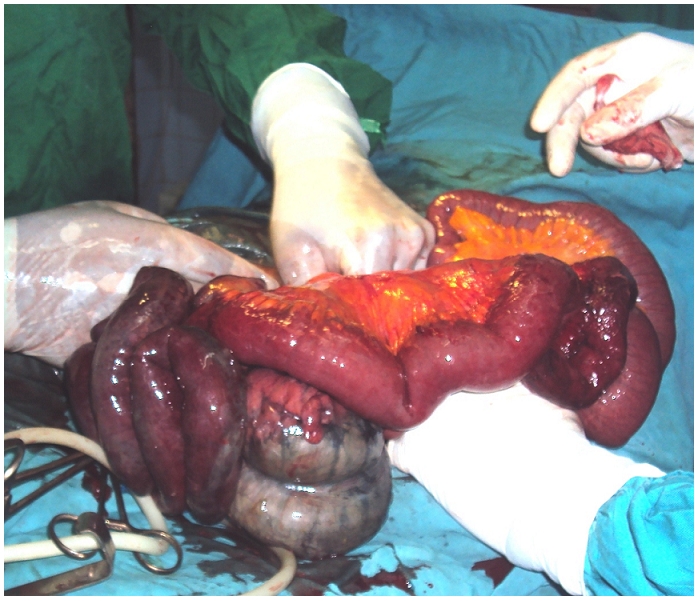
An intra-operative view of the abdomen of our patient showing extensive small bowel necrosis

**Figure 3:**
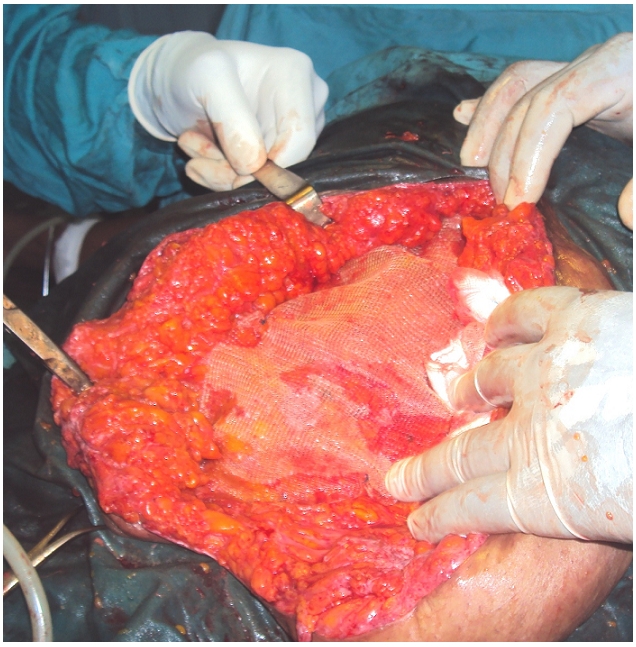
A view of the hernia repair. The non absorbable polypropylene mesh is placed pre-muscular, behind the rectus sheath

After a short period of resuscitation, a midline laparotomy is performed. Exploration shows a massive incisional hernia (hernia orifice is measured at 23 cm) with incarceration and necrosis of 135 cm of small bowel (Figure 2). Most of the jejunum and the final 80 cm of the ileum are spared. There is also free serous cloudy peritoneal fluid. A resection of the necrosed bowel is performed with end-to-end anastomosis. A pre-peritoneal 30x30 cm non absorbable polypropylene mesh is placed to repair the incisional hernia (Figure 3).

Immediate post-operative period is uneventful. On day 8th, the patient develops evidence of superficial wound infection with a fluctuating parietal collection. The diagnosis of an infected seroma is proposed. The collection is drained, samples are taken for identification and sensitivity and the antibiotic treatment adjusted accordingly. The drainage reduces gradually and stops completely after 16 days. The patient hasn't raised any new complain for the past six months.

## Discussion

Mesh repair remains the preferred treatment option for abdominal wall hernias, especially for large incisional hernias. Infection of an implanted mesh is the most feared complication of this type of repair and usually represents a major challenge. The incidence of mesh infection seems to depend on the approach. It has been estimated to be 1% with laparoscopic approach and up to 15% with open techniques [1–4].

In our patient, we deliberately decided to place a mesh in a context of high risk of infection. This was motivated by the need to have her problem of incisional hernia solved definitely. In our opinion, it wouldn't have been reasonable to perform a suture repair for an incisional hernia of 23 cm of diameter because of the fear of mesh infection. Very little is known on the outcome of mesh repair of incarcerated ventral hernias. The rate of infective complications was recently described to be increased by the need for bowel resection [3].

Until recently, mesh salvation was still considered rarely successful. Most cases of mesh infection would require excision and complex abdominal wall reconstruction with significant morbidity and mortality [2,5]. Recent findings in the literature have suggested different approaches to mesh salvation. These include daily diffusion of antiseptics, antibiotic irrigation and vacuum systems [1,6–8].

We proposed simple drainage to our patient. In a recent study, this procedure alone has proved to successfully salvage the mesh in 76% of cases, mostly polypropylene meshes [9].

## Conclusion

We conclude that it would be reasonable to consider mesh repair for incarcerated hernia. When infection occurs, a mesh salvaging approach can safely be considered.
